# Subcutaneous and Intraosseous Fat Necrosis Associated with Chronic Pancreatitis

**DOI:** 10.3390/medicina58060802

**Published:** 2022-06-14

**Authors:** Jelena D. Zivadinovic, Marko M. Stojanovic, Marija D. Stosic, Aleksandar R. Zivadinovic, Radmilo Jankovic, Marko D. Gmijovic, Ilija Golubovic, Biljana Stosic, Nebojsa S. Ignjatovic, Miroslav P. Stojanovic

**Affiliations:** 1 Clinic for Aneasthesiology and Intensive Therapy, University Clinical Center Nis, University of Nis, 18000 Nis, Serbia; marija91nis@gmail.com (M.D.S.); jankovic.radmilo@gmail.com (R.J.); b.stosic@yahoo.com (B.S.); 2 Gastroenterology and Hepatology Clinic, University Clinical Center Nis, University of Nis, 18000 Nis, Serbia; marcss994@gmail.com; 3 Clinic for Gynecology and Obstetrition, University Clinical Center Nis, University of Nis, 18000 Nis, Serbia; zivadinovicaleksandar92@gmail.com; 4 Digestive Surgery Clinic, University Clinical Center Nis, University of Nis, 18000 Nis, Serbia; markogmija@gmail.com (M.D.G.); golubovicilija@yahoo.com (I.G.); n.ignjat@gmail.com (N.S.I.); drmiroslavstojanovic@gmail.com (M.P.S.)

**Keywords:** subcutaneous, intraosseous fat necrosis, chronic pancreatitis

## Abstract

*Background:* Extra-abdominal manifestations of fat necrosis, like subcutaneous fat necrosis, polyarthritis, and polyserositis may appear with an occurrence rate of about 0.8%, wherein intraosseous fat necrosis is a more rare complication of pancreatitis, with few reports in English literature. *Case report:* A 34-year-old male with a 15-year-history of alcohol abuse was hospitalized several times in the last few years because of attacks of relapsed chronic pancreatitis. After the last attack, pancreatitis came in a stable state (“burned out”) with no symptoms and signs of the disease. The patient had been free of symptoms for 28 months since the last admission when he came with sub-febrile temperature, huge pain, swelling, and erythema in the area of the left lateral malleolar region with propagation in the foot. Blood biochemistry was normal. Conventional radiography showed multiple sites of osteolysis in the left calcaneus. Images on multislice computed tomography (MSCT) with 3D reconstruction revealed hypodense focuses that corresponded to osteonecrosis areas and bone marrow edema in the left calcaneus. *Conclusions:* The possibility of intraosseous fat necrosis should be considered in situations of unexplained polyarthritis or panniculitis, particularly in individuals with alcohol abuse or pancreatic disease.

## 1. Introduction

Peripancreatic, retroperitoneal, mesenteric and omental fat necrosis are frequent findings in acute and chronic pancreatitis [1]. Extra-abdominal manifestations of fat necrosis, like subcutaneous fat necrosis, polyarthritis and polyserositis may appear with an occurrence rate of about 0.8% [1,2], wherein intraosseous fat necrosis is a more rare complication of pancreatitis, with few reports in English literature [2,3,4,5,6,7,8].

Metastatic fat necrosis syndrome affects fewer than 1% of pancreatic disease patients [9]. It has been linked to acute or subacute alcoholic pancreatitis [2,7,10,11,12], traumatic pancreatitis [13], ischemia [14], congenital abnormalities [15] as well as pancreatic cancer [16,17,18,19]. The cutaneous panniculitis lesions have a preference for articular areas, resulting in periarticular inflammation [10]. Furthermore, lytic bone lesions, including meta-diaphyseal extremity regions, are thought to be caused by intraosseous fat necrosis caused by an excess of circulating lipase owing to pancreatitis. The triad of this syndrome consists of pancreatic disease, panniculitis and polyarthritis. Pancreatitis, panniculitis and polyarthritis syndrome (PPP syndrome) is the name given to this syndrome [20]. This syndrome occurs in fewer than 1% of patients with pancreatic diseases such as pancreatitis, pancreatic duct obstructions, pseudocysts, and pancreatic cancer [21,22], and it is more common in middle-aged males with a history of alcohol consumption and a history of acute pancreatitis relapses [23,24]. Dieker et al. [25] reported another 32 cases of PPP syndrome described in the literature in addition to their case report.

It is crucial to properly understand this disease in order to avoid misunderstanding or delayed identification of this uncommon complication. In this paper, a case of alcohol-induced chronic pancreatitis with subsequent massive unilateral subcutaneous and calcaneal intraosseous fat necrosis is reported, and this unusual complications are discussed based on the literature.

## 2. Case Report

A 34-year-old male, with a 15-years history of alcohol abuse (over 200 mL of pure ethanol daily) was hospitalized several times in the last few years because of attacks of relapsed chronic pancreatitis. Based on anamnesis, clear symptoms (pain, dyspepsia, weight loss and typical ultrasound, magnetic resonance cholangiopancreatography (MRCP) and (Computed tomography) CT findings of the “non-biliary small-duct” disease, the patient was treated on conservative manner, with strict alcohol restriction, diet, supportive enzymes as well as analgetics. Tumor markers levels were always within normal limits. Despite a good response to therapy, there were 4 moderate relapses in 3 years with pain aggravation, and raising of the amylase and lipase levels in blood and urine. After the last attack, pancreatitis came in a stable state, (“burned out”), with no symptoms and signs of the disease. Control MSCT showed almost normal shape, texture, vascular and canalicular system. 

**Figure 1:**
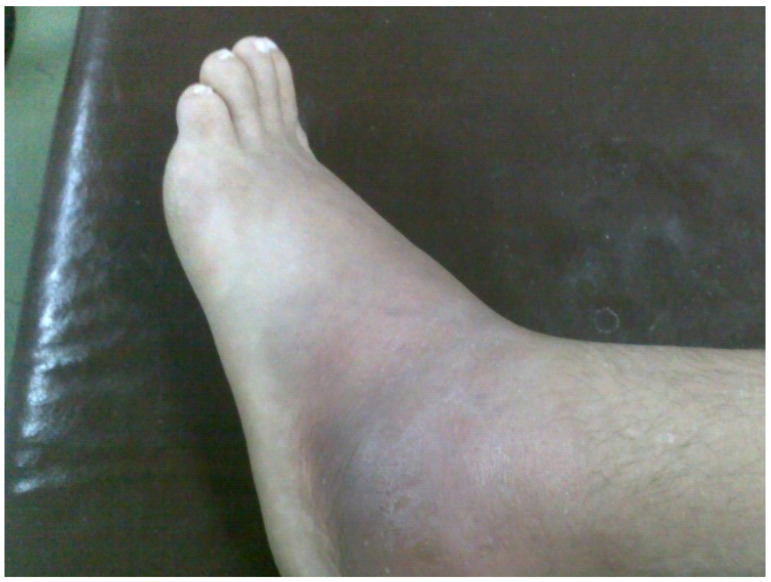
Swelling and erythema in the area of left lateral malleolar region with propagation in the foot.

The diet and supplements were continued and the patient was free of symptoms for 28 months after the last hospitalization when he came with sub-febrile temperature, huge pain, swelling, and erythema in the area of the left lateral malleolar region with propagation in the foot (Figure 1).

**Figure 2:**
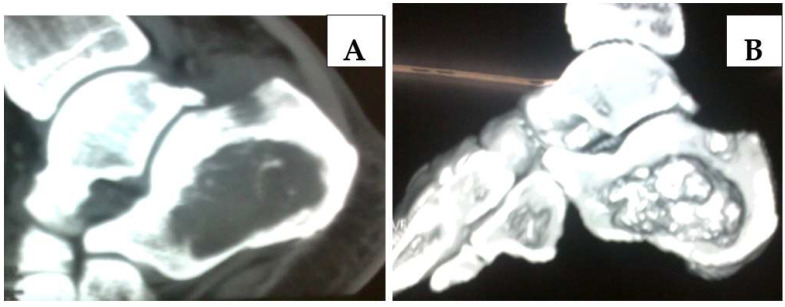
MSCT (**A**) with 3D reconstruction (**B**) revealed hypodense focuses of osteonecrosis and bone marrow edema in the left calcaneus. Intraarticular and soft-tissue edema were also noted.

Blood biochemistry (complete blood count, C-reactive protein- CRP, rheumatoid factor, amylase and lipase levels, hepatogram) was normal. Conventional radiography showed multiple sites of osteolysis in the left calcaneus. Images on MSCT (Figure 2A) with 3D reconstruction (Figure 2B) revealed hypodense focuses that correspond to osteonecrosis areas and bone marrow edema in the left calcaneus. This area within the marrow of the left calcaneus was compatible with a diagnosis of fat necrosis secondary to pancreatitis. Intraarticular and soft-tissue edema in the region of the talocrural joint was also noted. 

Percutaneous needle aspiration of the periarticular soft tissue was performed and a very small amount of the fluid was aspirated. Biochemical analysis showed elevated amylase (548 U/L), and lipase (1300 U/L) levels. There were not any signs of inflammatory exudate (white blood cells count, sugar, proteins, CRP, and interleukin-6were within the limits). Bacterial cultures were negative. An Orthopedic surgeon performed calcaneal curettage and mini-incisional drainage of subcutaneous lesions. Histopathological examination showed necrotic fat and connective tissue with moderate lymphocytes infiltration. 

The patient was initially treated surgically with calcaneal curettage and mini -incisional drainage of subcutaneous lesions. After which, nonsteroidal anti-inflammatory drugs (NSAID) and oral glucocorticoids were introduced. After 1 month, the symptoms and signs relating to the left ankle resolved. Six months after his last admission, calcaneal radiographs demonstrated a decrease in the osteolytic changes.

## 3. Discussion

Studies on this topic are limited to case reports and pathophysiology is undetermined. It is generally accepted theory that direct injury to the blood vessels by pancreatic enzymes and excessive release of circulating lipases in the course of pancreatitis results in fat necrosis and intramedullary lipolysis. Some researchers consider that inflammatory response and necrosis in articular areas are caused by free fatty acids which are formed by the hydrolysis of triglycerides under the action of pancreatic lipases [26]. Intra-abdominal fat necrosis of the retroperitoneum, mesentery, and omentum is a frequent complication of acute, chronic, and traumatic pancreatitis [1]. Extra-abdominal forms of fat necrosis, such as subcutaneous fat necrosis, polyarthritis, polyserositis, and intraosseous fat necrosis, can develop at an incidence of around 0.8 percent [1,2]. Subcutaneous fat necrosis -panniculitis lesions are more common. Predilected sites for panniculitis lesions are articular regions with subsequent periarticular inflammation and may be associated with synovitis. The ankle is the most commonly involved joint [27] as we registered in our case. Bone marrow involvement in acute pancreatitis was first described by Ponfick in 1872 [28]. It has also been associated with pancreatic carcinoma [29], ischemia [14], and congenital abnormalities [16]. Osteolytic changes are more often in the course of traumatic pancreatitis with the onset 3 to 4 weeks after the initial episode [6]. Very rarely, articular symptoms can occur before pancreatic disease is diagnosed [30]. In our case, the skin and bone lesions developed 2 years following the diagnosis of chronic pancreatitis, with no aggravation of the pancreatic disease during this period. Intraosseous fat necrosis often affects long bones of the extremities, with few reports of the affected vertebra. Although the data from Obatake [28], Keating [31] and Boswel [32] suggest that osteolytic lesions dominantly occur symmetrically and at the metaphyses of long bones, there is no regularity in the number and localization of the osteolytic lesions, with slight predominant of multiplicity [3,4,27,32] despite our case report of strictly unilateral calcaneal involvement.

The clinical symptoms include a febrile state (or not), pain in multiple joints, swelling, and erythema in the affected areas. This syndrome is known as PPP syndrome (pancreatic disease, panniculitis and polyarthritis) [20] and was first described in 1908 by Berner [26]. A 2017 study by Dieker et al. reported another 32 cases of this syndrome described in the literature in addition to their case report [25]. The vast majority of patients, as well as our patient, didn’t have abdominal symptoms, which may lead to misdiagnosis [30].

Conventional radiography shows multiple sites of osteolysis and correlates pathologically with areas of intramedullary fat necrosis and trabecular bone destruction [3]. Over time, diagnostic features of osteonecrosis with resultant bone sclerosis develop, often progressing to fragmentation and subsequent subarticular bone collapse. There is increased uptake of technetium pyrophosphate scintigraphy in pathological areas [2,28]. Images on magnetic resonance imaging (MRI) show low signal foci on T-1 weighted images and heterogeneous or low signal loci on T-2 weighted images that correspond to osteonecrosis areas and bone marrow edema [2]. In our case, we performed MSCT with a 3D reconstruction which showed hypodense focuses that correspond to osteonecrosis areas and bone marrow edema in the left calcaneus. This area within the marrow of the left calcaneus was compatible with a diagnosis of fat necrosis secondary to pancreatitis. There was also intraarticular and soft-tissue edema in the talocrural joint area. 

Metastatic bone tumors and osteomyelitis are the differential diagnoses, and diagnosis is made based on histological and bacterial culture tests [6]. It is also important to consider avascular necrosis, especially non-traumatic avascular necrosis associated with alcohol consumption [33]. The diagnosis is made based on the anamnesis, clinical symptoms of pancreatitis and elevated levels of pancreatic enzymes in synovial fluid. We have demonstrated elevated levels of lipase and amylase in the periarticular fluid collection. Also, Loverdos et al. detected elevated levels of lipase in the peripheral fluid collection at the site of affected fingers [34] and Simkin et al. detected elevated levels of lipase in the synovial fluid [12].

The radiological changes remain for several months after the improvement of clinical symptoms [28]. In our case, the bone lesions developed 2 years following the diagnosis of chronic pancreatitis with the stable pancreatic disease at that moment and resolved after calcaneal curettage and mini -incisional drainage of subcutaneous lesions. 

## 4. Conclusions

In conclusion, we reported a rare case of subcutaneous and intraosseous fat necrosis associated with chronic alcoholic pancreatitis. The possibility of intraosseous fat necrosis should be considered in situations of unexplained polyarthritis or panniculitis, particularly in individuals with alcohol abuse or pancreatic disease.

## Data Availability

Not applicable.

## References

[1] Čolović R., Grubor N., Radak V., Čolović N., Ranković V., Latinčić S., Matić S. (2008). Disseminated subcutaneous fat necrosis and elbow joint arthritis as a complication of pancreatitis. VSP.

[2] Haller J., Greenway G., Resnick D., Kindynis P., Kang H.S. (1989). Intraosseous fat necrosis associated with acute pancreatitis: MR imaging. Radiology.

[3] Karasick D., Schweitzer M.E. (1998). Case 4: Intraosseous, fat necrosis associated with pancreatitis. Radiology.

[4] Kotilainen P., Saario R., Mattila K., Nylamo E., Aho H. (1998). Intraosseous fat necrosis simulating septic arthritis and osteomyelitis in a patient with chronic pancreatitis. Arch. Orthop. Trauma Surg..

[5] Morita O., Ogose A., Hotta T., Kawashima H., Higuchi T., Suzuki K., Endo N. (2003). Pathological fractures due to intraosseous fat necrosis associated with pancretitis. Reumatology.

[6] Baba T., Shitoto K., Yoshioka C., Kaneko H. (2011). Pathological fracture due to vertebral osteonecrosis associated with pancreatitis. Arch. Orthop. Trauma Surg..

[7] Norimura D., Mizuta Y., Ohba K., Oh J., Oohara H., Nakahara N., Yamaguchi N., Ohnita K., Isomoto H., Shikuwa S. (2009). Intraosseous fat necrosis associated with alcoholic pancreatitis. Clin. J. Gastroenterol..

[8] Tupalli A., Angamuthu M., Prashanth A., Singh R., Kumar R. (2020). Intraosseous Medullary Fat Necrosis on 99mTc-MDP Bone Scan of Patient With Acute Pancreatitis. Clin. Nucl. Med..

[9] Tannenbaum H., Anderson L.G., Schur P.H. (1975). Association of polyarthritis, subcutaneous nodules, and pancreatic disease. J. Rheumatol..

[10] Kushner D.S., Szanto P.B. (1958). Fulminant polyarthritis, fever, and cutaneous nodules in an alcoholic patient. J. Am. Med. Assoc..

[11] Kim E.J., Chu M.S., Sohn K.C., Cho D.H., Na G.H., Kim H.C., Cho E.Y. (2017). Pancreatic Panniculitis in Patients with Chronic Pancreatitis: Case Report and Review of Literature. Korean J. Gastroenterol..

[12] Simkin P.A., Brunzell J.D., Wisner D., Fiechtner J.J., Carlin J.S., Willkens R.F. (1983). Free fatty acids in the pancreatitic arthritis syndrome. Arthritis Rheum..

[13] Schutte H.E., Wackwitz J.D. (1981). Case report 171: Metastatic fat necrosis involving the tubular bones of the hands (and probably the feet) secondary to traumatic pancreatitis. Skelet. Radiol..

[14] Smukler N.M., Schumacher H.R., Pascual E., Brown S., Ryan W.E., Sadeghian M.R. (1979). Synovial fat necrosis associated with ischemic pancreatic disease. Arthritis Rheum..

[15] Haber R.M., Assaad D.M. (1986). Panniculitis associated with a pancreas divisum. J. Am. Acad. Dermatol..

[16] Virshup A.M., Sliwinski A.J. (1973). Polyarthritis and subcutaneous nodules associated with carcinoma of the pancreas. Arthritis Rheum..

[17] Radin D.R., Colletti P.M., Forrester D.M., Tang W.W. (1986). Pancreatic acinar cell carcinoma with subcutaneous and intraosseous fat necrosis. Radiology.

[18] Takeuchi Y. (2020). Pancreatitis, panniculitis, and polyarthritis syndrome complicated with terminal pancreatic adenocarcinoma managed with intra-articular knee aspiration, intra-articular lidocaine and corticosteroid injection, and decompression of panniculitis: A case report. J. Gen. Fam. Med..

[19] Zhang G., Cao Z., Yang G., Wu W., Zhang T., Zhao Y. (2016). Pancreatic panniculitis associated with pancreatic carcinoma: A case report. Medicine.

[20] Narváez J., Bianchi M.M., Santo P., de la Fuente D., Ríos-Rodriguez V., Bolao F., Narváez J.A., Nolla J.M. (2010). Pancreatitis, panniculitis, and polyarthritis. Semin. Arthritis Rheum..

[21] Watts R.A., Kelly S., Hacking J.C., Lomas D., Hazleman B.L. (1993). Fat necrosis. An unusual cause of polyarthritis. J. Rheumatol..

[22] Ferrari R., Wendelboe M., Ford P.M., Corbett W.E., Anastassiades T.P. (1993). Pancreatitis arthritis with periarticular fat necrosis. J. Rheumatol..

[23] Immelman E., Bank S., Krige H. (1964). Roentgenologic and clinical features of intramedullary fat necrosis in bones in acute and chronic pancreatitis. Am. J. Med..

[24] Phillips R.M., Sulser R.E., Songcharoen S. (1980). Inflammatory arthritis and subcutaneous fat necrosis associated with acute and chronic pancreatitis. Arthritis Rheum..

[25] Dieker W., Derer J., Henzler T., Schneider A., Rückert F., Wilhelm T.J., Krüger B. (2017). Pancreatitis, panniculitis and polyarthritis (PPP-) syndrome caused by post-pancreatitis pseudocyst with mesenteric fistula. Diagnosis and successful surgical treatment. Case report and review of literature. Int. J. Surg. Case Rep..

[26] Castro J.P., Atanásio G., Canelas M.A., Ferreira A., Barbosa A.R., Barbedo M., Abreu R. (2020). A case report of pancreatitis-panniculitis-polyarthritis syndrome-An unusual but serious presentation of pancreatic disease. Scott. Med. J..

[27] Rodriguez M., Lopez G.L., Prieto P., Fernandez L., Willisch A., Arce M. (1997). Massive Subcutaneous and Intraosseous Fat Necrosis Associated with Pancreatitis. Natural Evolution of the Radiographic Picture. Clin. Rheumatol..

[28] Obatake M., Yamane Y., Tokunaga T., Taura Y., Inamura Y., Nagayasu T. (2009). Arthralgia and Osteolytic Lesions Associated with Traumatic Pancreatitis in a 10-Year-Old Girl. Int. J. Pediatrics.

[29] Arbeláez-Cortés A., Vanegas-García A.L., Restrepo-Escobar M., Correa-Londoño L.A., González-Naranjo L.A. (2014). Polyarthritis and pancreatic panniculitis associated with pancreatic carcinoma: Review of the literature. J. Clin. Rheumatol..

[30] Langenhan R., Reimers N., Probst A. (2016). Osteomyelitis: A rare complication of pancreatitis and PPP-syndrome. Jt. Bone Spine.

[31] Keating J.P., Shackelford G.D., Shackelford P.G., Ternberg J.L. (1972). Pancreatitis and osteolytic lesions. J. Pediatrics.

[32] Boswell S.H., Baylin G.J. (1973). Metastatic fat necrosis and lytic lesions in a patient with painless pancreatitis. Radiology.

[33] Callachand F., Milligan D., Wilson A. (2016). Atraumatic Pantalar Avascular Necrosis in a Patient with Alcohol Dependence. J. Foot Ankle Surg..

[34] Loverdos I., Swan M.C., Shekherdimian S., Al-Rasheed A.A., Schneider R., Fish J.S., Ngan B.Y., Adeli K., Lowe M.E., Singh V.P. (2015). A case of pancreatitis, panniculitis and polyarthritis syndrome: Elucidating the pathophysiologic mechanisms of a rare condition. J. Pediatrics Surg. Case Rep..

